# Artificial Intelligence to Close the Gap between Pharmacokinetic/Pharmacodynamic Targets and Clinical Outcomes in Critically Ill Patients: A Narrative Review on Beta Lactams

**DOI:** 10.3390/antibiotics13090853

**Published:** 2024-09-06

**Authors:** João Gonçalves Pereira, Joana Fernandes, Tânia Mendes, Filipe André Gonzalez, Susana M. Fernandes

**Affiliations:** 1 Grupo de Investigação e Desenvolvimento em Infeção e Sépsis, Clínica Universitária de Medicina Intensiva, Faculdade de Medicina, Universidade de Lisboa, 1649-004 Lisbon, Portugal; 2 Serviço de Medicina Intensiva, Hospital Vila Franca de Xira, 2600-009 Vila Franca de Xira, Portugal; 3 Grupo de Investigação e Desenvolvimento em Infeção e Sépsis, Serviço de Medicina Intensiva, Centro Hospitalar de Trás-os-Montes e Alto Douro, 5000-508 Vila Real, Portugal; jcjfernandes@chtmad.min-saude.pt; 4 Serviço de Medicina Interna, Hospital Vila Franca de Xira, 2600-009 Vila Franca de Xira, Portugal; tania.mendes@ulsetejo.min-saude.pt; 5 Serviço de Medicina Intensiva, Hospital Garcia De Orta, Clínica Universitária de Medicina Intensiva, Faculdade de Medicina, Universidade de Lisboa, 1649-004 Lisbon, Portugal; filipe.gonzalez@ulsas.min-saude.pt; 6 Grupo de Investigação e Desenvolvimento em Infeção e Sépsis, Serviço de Medicina Intensiva, Hospital Santa Maria, Clínica Universitária de Medicina Intensiva, Faculdade de Medicina, Universidade de Lisboa, 1649-004 Lisbon, Portugal; susanamfernandes@medicina.ulisboa.pt

**Keywords:** β-lactams, antibiotic concentrations, therapeutic drug monitoring, pharmacodynamics, biomarkers, machine learning

## Abstract

Antimicrobial dosing can be a complex challenge. Although a solid rationale exists for a link between antibiotic exposure and outcome, conflicting data suggest a poor correlation between pharmacokinetic/pharmacodynamic targets and infection control. Different reasons may lead to this discrepancy: poor tissue penetration by β-lactams due to inflammation and inadequate tissue perfusion; different bacterial response to antibiotics and biofilms; heterogeneity of the host’s immune response and drug metabolism; bacterial tolerance and acquisition of resistance during therapy. Consequently, either a fixed dose of antibiotics or a fixed target concentration may be doomed to fail. The role of biomarkers in understanding and monitoring host response to infection is also incompletely defined. Nowadays, with the ever-growing stream of data collected in hospitals, utilizing the most efficient analytical tools may lead to better personalization of therapy. The rise of artificial intelligence and machine learning has allowed large amounts of data to be rapidly accessed and analyzed. These unsupervised learning models can apprehend the data structure and identify homogeneous subgroups, facilitating the individualization of medical interventions. This review aims to discuss the challenges of β-lactam dosing, focusing on its pharmacodynamics and the new challenges and opportunities arising from integrating machine learning algorithms to personalize patient treatment.

## 1. Introduction

Precision antimicrobial dosing has two different goals: improving the outcomes of infectious diseases while minimizing toxicity and preventing the emergence of antimicrobial resistance [1]. Artificial intelligence and software development with pharmacokinetic/pharmacodynamic (PK/PD) models from critically ill patients can contribute to the individualization of β-lactam dosage and administration regimen. 

Optimization of antimicrobial prescription in critically ill patients is challenging due to unpredictable inter and intraindividual PK variability [2,3]. Sepsis-related pathophysiological changes, organ failure, and therapeutic interventions influence antibiotic concentration in septic patients [3,4]. Target concentrations of β-lactam antibiotics, the most administered antibiotic in critically ill patients, are difficult to achieve using standard dosing [2,3]. Evidence shows that up to 50% of patients receiving these antimicrobials fail to achieve their ideal PK/PD target. This is associated with a lower probability of clinical cure and bacteriological eradication and promotes the development of antimicrobial resistance [2,5,6]. Due to PK heterogeneity in critically ill patients, during the treatment period, real-time adjustments of the antibiotic dose to the patient’s condition are critical [3]. The percentage of the time that the antibiotic-free concentration exceeds the minimum inhibitory concentration (% fT > MIC) of 40–70% is the PK/PD index generically associated with optimal β-lactam activity [7]. However, data from critically ill populations suggests that longer and higher β-lactam exposures than those described in in vitro and in vivo animal model studies might be beneficial [2,8,9,10,11]. A PK/PD target of a 100% fT > 4 × MIC plasma concentration has been proposed to maximize bacteriological and clinical responses [9,10,11,12].

A current approach to optimize β-lactam exposure is to use higher-than-standard doses in the initial phase of sepsis, to counterbalance the increased volume of distribution, especially during the first 48 h, and guide subsequent dosing according to drug clearance, mainly by renal and hepatic function [3,13]. Another strategy is to administer an initial intravenous loading dose, at the onset of treatment, followed by prolonged (continuous or extended) β-lactam infusion to maximize PK/PD target attainment [7,12,14]. The use of prolonged administration of β-lactams is particularly relevant in patients with sepsis or septic shock, in the case of bacteria with a high MIC, in non-fermenting Gram-negative bacteria, and in lower respiratory tract infections, to improve clinical cure [12,15]. An additional and personalized intervention to optimize PK/PD targets is therapeutic drug monitoring (TDM)-guided therapy, but its clinical benefit remains a topic under debate. Notwithstanding, an international panel of experts, in a position paper, recommended routine TDM to be performed for β-lactam antibiotics in critically ill patients, to minimize toxicity and optimize dosing [7]. Few randomized clinical trials have studied the effect of TDM on clinical outcomes, especially in patients with the most severe sepsis. A recently published meta-analysis of five different studies involving 1011 critically ill patients showed no significant benefit in 28-day mortality, hospital mortality, length of intensive care unit (ICU) stay, or clinical cure with a TDM-based regimen [16]. Additionally, this systematic review did not support the results of previous trials describing an increase in target attainment when applying TDM [17,18,19,20,21].

An emerging method for TDM-guided dosing is model-informed precision dosing (MIPD), which uses pharmacometrics modeling to estimate future antibiotic exposure. MIPD is based on population PK models and TDM results for steady-state concentrations. However, the first multicenter study addressing the MIPD effect on clinical outcomes, published recently, including in both arms roughly 20% of patients in septic shock, did not find a significant difference either in length of ICU stay or target attainment between early MIPD and standard dosing [22]. Intra and inter-individual PK variability in critically ill patients (especially those with sepsis and septic shock) creates additional difficulties in the use of MIPD. Furthermore, differences in bacteria and infection foci have scarcely been studied but have a potential role in impacting antibiotic PD [23].

In this review, we address the impact of PK and PD use on clinical outcomes, as well as its limitations. We discuss the potential benefits of the integration of clinical, biomarker, and microbiological data using artificial intelligence (AI) models to adjust antibiotic dosing.

### Impact of Therapeutic Drug Monitoring on the Outcomes

**Figure 1:**
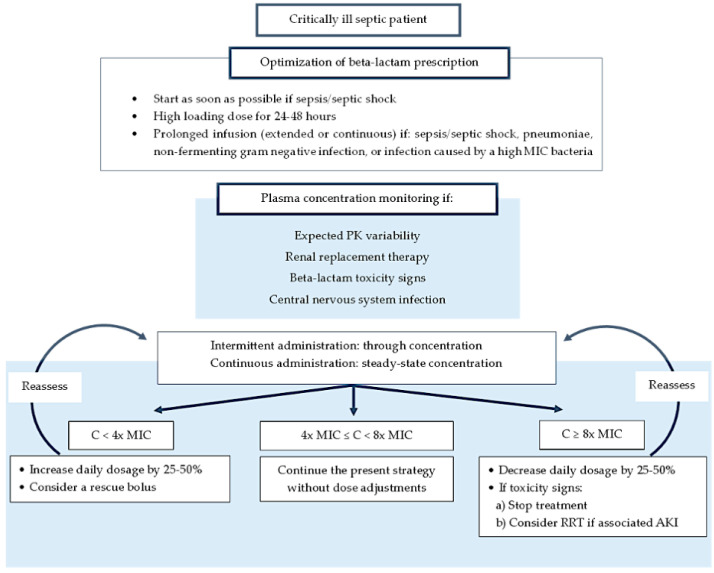
A practical algorithm based on β-lactam dose optimization by Therapeutic Drug Monitoring (adapted from references [12,15]). PK–Pharmacokinetics; C—plasma concentration; MIC—minimum inhibitory concentration; RRT—renal replacement therapy; AKI—acute kidney injury.

Although no study has shown the impact of TDM on outcomes, the principles that support its use in critically ill patients must be addressed. TDM is particularly recommended [12] in critically ill patients in the following situations: (a) patients with expected significant β-lactam PK variability; (b) patients with clinical signs potentially related to β-lactam toxicity; (c) patients undergoing renal replacement therapy; (d) patients with a central nervous system infection (blood and cerebrospinal fluid samples collected simultaneously are recommended). A practical algorithm based on β-lactam dose optimization by TDM can be found in Figure 1.

Possible explanations for the described discrepancy between theoretical achievement and outcomes include the following: (a) most trials having been restricted to a limited number of antibiotic types; (b) the inclusion of non-critically ill patients; c) heterogeneity in infection severity and lack of subgroup analysis; (c) blood serum examination, which might not have the same concentrations as the site of infection; (d) different dosing plasma concentration/target attainment measurement methods and timings (spotting differences in intermittent or continuous administration); (e) variable first antibiotic exposure (recognizing the relationship between early antibiotic exposure and patient outcomes); (f) unspecified first-loading dose (or dose not adjusted to organ dysfunction during the first 48 h), which may be suboptimal; and (g) delay in dose adaptation arising from assay turnover time [18,20,21]. To complicate things further, the diagnosis of infection is often flawed [24], even when microbiologically documented (no test can preclude the existence of colonization). More consistent protocols and outcome settings across studies may clarify the beneficial clinical effect of TDM-based antibiotic regimens.

## 2. Reasons for the Absence of Response Despite Adequate Plasma Concentrations 

Antimicrobial dosing aims to achieve maximum bacterial killing without causing harm to host cells. Although in vitro evidence supports a strong relationship between antibiotic exposure and bacterial killing, there is still a lack of clinical evidence to support the use of specific therapeutic targets [3]. The variability of antibiotic PK between subjects, and within subjects, during therapy is well-known [25,26].

Knowledge of PK/PD targets is poor and mostly based on observational data. Moreover, only plasma antibiotic concentrations support pharmacokinetic studies, which may be misleading since infection occurs in tissues [27,28]. Conflicting data suggest a poor correlation between the different suggested PK/PD targets and the achievement of infection control.

Different reasons may contribute to this discrepancy:

1. β-lactam tissue penetration may be influenced by tissue perfusion, adequate microcirculation availability, tissue inflammation, or necrosis;
2. the PD value, which mainly reflects the antibiotic time-kill kinetics, is strongly influenced by the phenotypical expression of bacteria, namely the presence of biofilms;
3. bacterial tolerance and acquisition of resistance during therapy may lead to poor bacterial clearance and therapeutic failure.

Consequently, either a fixed dose of antibiotics or a fixed target concentration (achieved by therapeutic drug monitoring) may not provide the desired outcomes [29,30].

Increasing the dosage to achieve adequate killing of bacteria is tempting but may lead to several undesired toxic effects and dysbiosis [3].

### 2.1. Tissue Penetration

Tissue penetration with β-lactam antibiotics is strongly influenced by critical disease and its related PK changes [31]. Consequently, tissue concentrations may be harder to predict than unbound plasma concentrations [32].

Highly variable tissue concentration has been described, with large interindividual variability. Moreover, there is also within-patient variability because of fluctuations in inflammation. Off-label dosing regimens have been proposed as a potential solution under particular conditions [33], although toxicity may be a limitation.

This may be important since most infections occur in different body compartments and hypoperfusion may be significant in sepsis [34].

**Figure 2:**
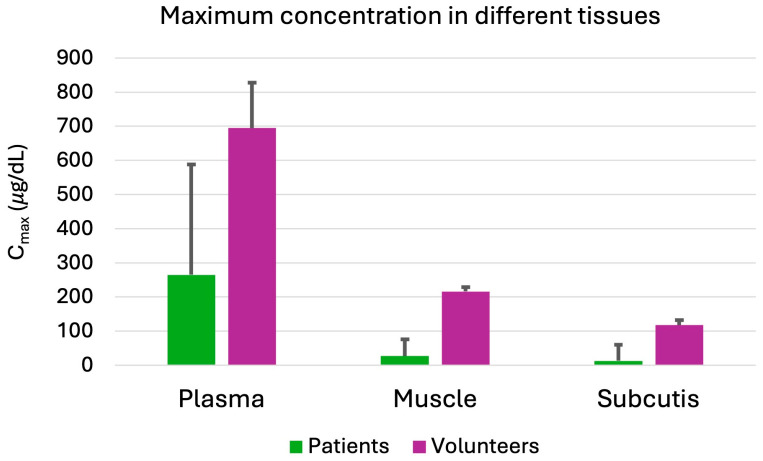
Maximum concentration in different tissues. Bars depict means and SD. In critically ill patients, the plasma concentrations are lower than in volunteers. Also, the ratios of tissue to plasma concentrations are reduced. Differences between volunteers and patients *p* < 0.005; muscle and subcutis tissue: differences between volunteers and patients *p* < 0.05. Data from reference [42].

Low antibiotic concentrations in critically ill patients have been described in lung epithelial line fluid [35,36,37,38], burn tissue [39], subcutaneous tissue [40,41,42], muscle [42,43] (Figure 2), and peritoneum [44,45] of infected patients. Nevertheless, a clear relationship between antibiotic tissue concentration and outcomes is lacking [27].

Since tissue PK assessment is not easily feasible, antibiotic treatment should be tailored according to the specific patient’s pathophysiological processes. Biomarkers assessing inflammation, endothelial function, and coagulation can guide antibiotic dosing, particularly in difficult-to-treat patients [46].

This personalized approach is useful to identify the populations more prone to benefit from direct applications of antibiotics, e.g., aerosolized antibiotics [47].

### 2.2. Biofilms

Cells within a microbial community can sense changes in the population density and adjust the expression of their genes accordingly [48]. The cell-to-cell communication mechanism is known as “quorum sensing”, which refers to the ability of cells to “sense” the presence of mediators produced by all bacteria. The density of mediators reflects the number of cells in the population (“quorum”). Accordingly, when this density reaches a threshold, quorum sense-signaling promotes collective phenotype switching [49]. 

Phenotype changes can lead to the self-secretion of a hydrated matrix composed of polysaccharides, proteins, water, and nucleic acids, responsible for biofilms [50]. These communities can be homogeneous but are often polymicrobial and incorporate part of the host organism [50].

Microbial biofilms can colonize medical devices and human tissues, especially when these are damaged. Their role in microbial pathogenesis is well established and is estimated to be associated with approximately two-thirds of nosocomial infections [51]. Biofilms provide resistance against antimicrobials and host defenses, promoting difficult-to-eradicate infections [51,52].

The differences between bacteria in a biofilm versus the planktonic (free-floating) phenotype are striking. Biofilm cells have modified doubling times and present reduced susceptibility to antimicrobials [50].

Biofilms provide bacteria with resistance mechanisms to antibiotics, namely their neutralization by the polysaccharide matrix, extracellular β-lactamases, their inactivation mediated by the combined activation of resistance genes, and plasmid-mediated transfer of resistance. Antibiotic susceptibility may differ in these bacteria cells compared with those evaluated in vitro [53]. Social protection, that is, the ability of β-lactamase-producing bacteria to protect others, may also occur in biofilms [54] and further contributes to antibiotic failure in vivo. Consequently, different patterns of antibiotic activity may be found and higher-than-predicted β-lactam concentrations may be needed [55].

### 2.3. Acquisition of Resistance: Bet-Hedging

Bacterial killing results from a complex interaction between antibiotics, their concentration, their ability to reach therapeutic targets, and the effect of the target–antibiotic complex on bacterial function. Small mutations in bacteria related to the therapeutic target may influence antibiotic susceptibility [56] but not necessarily cause resistance.

Bacterial communities are continuously interacting with the environment and adapting to its evolution. Accordingly, small mutations in different proteins may allow ready switching of phenotypes, resulting in different subpopulations that can adapt to various environmental challenges [49]. A particular case of phenotypic heterogeneity has been defined as bet-hedging, a risk-spreading strategy where bacteria present different phenotypes in the same population. It is essential to understand that this differentiation of phenotypes often results in some sub-populations maladapted to each environment, which have lower fitness and reproductive kinetics. However, this trade-off may provide the whole bacterial population with a selective advantage upon sudden environmental shifts, which may occur under therapeutical circumstances (e.g., exposure to antibiotics) [57].

It is unknown whether physiological parameters influence this kind of cellular differentiation process or whether it is purely a stochastic phenomenon. In a model of Bacillus subtilis, the bacterial population presented two distinct subpopulations: sporulating and nonsporulating cells. The physiological state of the cells’ ancestors strongly influenced cellular differentiation, unveiling an “epigenetic inheritance” [58].

**Figure 3:**
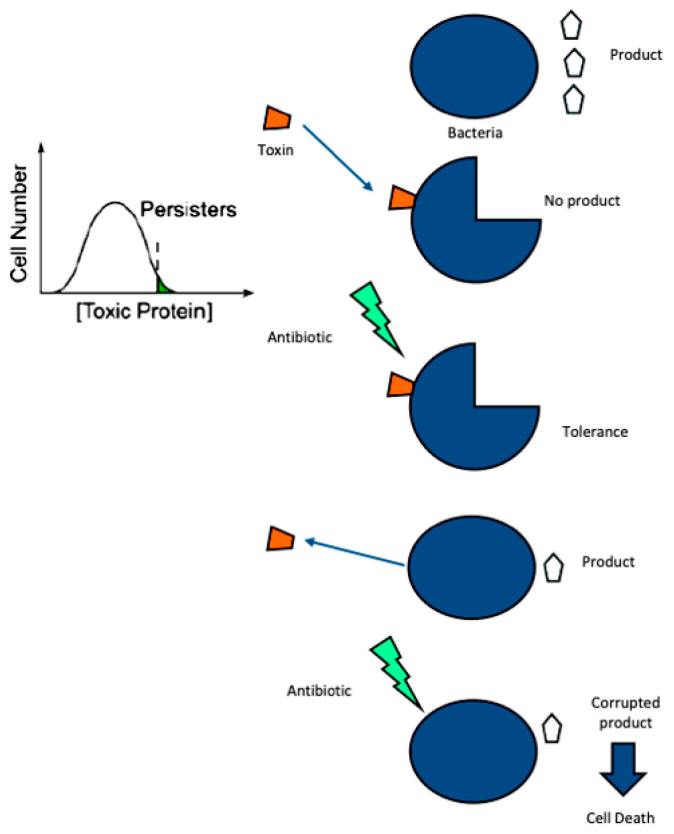
Persister cells. The antibiotic target is blocked and not functioning in a small proportion of bacteria. This leads to antibiotic failure since the antibiotic can no longer reach its receptor. This is different from resistance. After removal of the blockage, bacteria restart their normal functions and are again susceptible to antibiotics.

A specific type of bet-hedging is the ability of bacterial cell populations to overexpress many genes (over 300) that lead bacteria to “shut down” specific functions, like replication, translation, or modulation of proteins [59]. This phenomenon, called persister cells, resists many toxic substances (like antibiotics) but does not promote bacterial growth. Under appropriate circumstances, like antibiotic removal, persister cells can restart their metabolism and reduce these genes’ expression (Figure 3). The resultant bacteria have the same phenotypic expression as their ancestors [59].

### 2.4. Acquisition of Resistance during Therapy

Clinically relevant bacterial resistance to antibiotics poses a significant challenge to modern medicine. This resistance, often acquired during antibiotic therapy, undermines the efficacy of treatments, leading to persistent infections and increased mortality rates [60,61].

**Figure 4:**
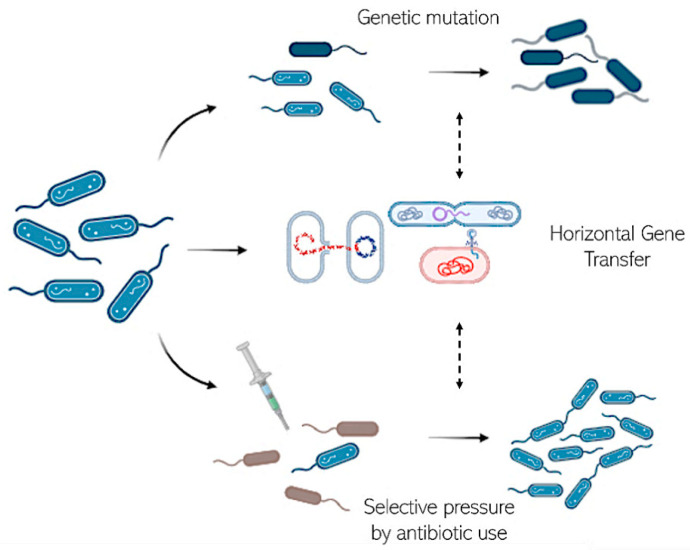
Acquisition of bacterial resistance through various mechanisms: genetic mutation, horizontal gene transfer, and selective pressure exerted by antibiotic use. Created in BioRender.com with permission.

Bacteria can acquire resistance through various mechanisms, including genetic mutation, horizontal gene transfer, and selective pressure exerted by antibiotic use (Figure 4). Spontaneous genetic mutations can confer resistance by altering the target site of the antibiotic, reducing drug uptake, or increasing drug efflux [62].

Horizontal gene transfer between bacteria facilitates the rapid dissemination of resistance genes across different bacterial species. Plasmids, transposons, and integrons often carry multiple resistance genes, promoting the development of multi-drug-resistant strains [63].

The selective pressure created by antibiotic therapy can favor the survival and proliferation of resistant bacteria, especially when suboptimal dosing, incomplete courses of treatment, and the use of broad-spectrum antibiotics come into play [64]. 

The gut microbiome plays a pivotal role in the emergence of antibiotic resistance. Antibiotic use facilitates the selection of resistant bacteria in the gut, which can multiply due to decreased competition and through the induction of resistance mechanisms [65].

Several factors contribute to the acquisition and spread of bacterial resistance during therapy. Inappropriate use of antibiotics, such as over-prescription and misuse for viral infections or without proper diagnosis, significantly drives resistance [60]. Additionally, failure to adhere to the scheduled prescription of the antibiotic course can foster selection and propagation of resistance [61]. The hospital environment, with its high antibiotic use and vulnerable patient populations, acts as a hotspot for antibiotic-resistant bacteria, especially when infection control practices are inadequate [60]. 

Addressing antibiotic resistance requires a multifaceted approach. Rational antibiotic prescribing, guided by susceptibility testing and informed by the minimum inhibitory and the mutant-prevention concentrations, ensures that patients receive the most effective treatment with the minimum risk of promoting resistance [66]. Also, recent research has highlighted the importance of optimal dosing strategies in critical patients to avoid resistance and toxicity [3].

The use of AI to improve the reporting of antibiograms has been proposed [67,68] and may enhance antibiotic stewardship programs [67], especially the reduction of broad-spectrum antibiotic use. Integration of antibiograms with patient data may create a unique opportunity to provide individualized, real-time recommendations [69] and improve outcomes.

### 2.5. Compensatory Mutations

Antibiotic exposure may induce bacterial mutagenesis or facilitate the acquisition of resistance mechanisms by lateral transfer. The environment is likely to influence the relative importance of these mechanisms [70]. However, there is a fitness cost to the changes in gene expression that lead to antibiotic resistance, and, in the absence of antibiotic exposure, this may slow or reverse. The changes induced by the exposure of the gut microbiome to antibiotics may reverse to near-baseline composition within 1.5 months [71,72], although the time to recover may be influenced by age [73]. Nevertheless, common microbiome species may disappear and remain undetectable, even after longer periods [71,72].

Compensatory mutations correct the loss of fitness due to earlier mutations. They lower the fitness cost and may lead to entrenched resistance. Accordingly, the early institution of combination antibiotic therapy may be necessary to overcome this mechanism [74], which may facilitate the persistence of plasmids conferring resistance to diverse antibiotics [75]. Slow and dynamic changes in target genes between compensatory and neutral mutations may lead to the development and predominance of these new fit, resistant microorganisms, even after exposure to the promoting antibiotic [76].

## 3. Therapeutic Response to β-Lactams to Guide Antibiotic Dosing

Several questions must be answered in the days after the antibiotic prescription: Is the antibiotic adequate according to isolates in cultures? Is the host recovering? For how long should the antibiotic be maintained?

The answer to the first question depends on microorganism isolation, the determination of minimal inhibitory concentrations, and the evaluation of antibiotic concentrations, as was previously discussed. Monitoring therapeutic response to β-lactams also relies on host improvement, which is evaluated through the resolution of the inflammatory response and recovery of the functions of the affected organs. The resolution of the inflammatory response is based on clinical response and evaluation of biomarkers.

### 3.1. Clinical Response and Monitoring

The host response to infection relies on temperature and metabolic demand changes, leading to an increasing cardiac output. The evaluation of recovery after antibiotic prescription can be based on temperature normalization, heart and respiratory rate stabilization for severe cases, and an overall improvement in well-being. If there is a fever or hypothermia, temperature normalization is expected to occur within the first 48–72 h. Nevertheless, there is very little evidence to predict the exact moment when temperature shifts are no longer expected if there is an adequate response to antibiotics. In infections resistant to antimicrobial drugs, recovery might be delayed even in community settings [77]. 

In addition to temperature, heart, and respiratory rate regularization, the symptoms associated with the infection must also subside for a complete clinical response to be declared. Different response rates are expected according to the infection site. Clinical resolution for urinary tract symptoms in men is expected to be complete in the first seven days [78]. In women, symptom resolution can occur after the first day of antibiotic treatment. In a randomized control trial comparing two different β-lactams in complicated urinary tract infections, more than 98% of patients achieved clinical and microbiological cure at 28 days [79].

Recently, in a randomized control trial evaluating non-severe respiratory infection treated in the ambulatory setting (90% of patients treated with amoxicillin with or without clavulanate), early antibiotic termination, based only on clinical symptoms, was associated with reduced use of antimicrobial drugs and no increase in adverse events [80]. For severe respiratory infections, symptoms can take longer to resolve, even if the antibiotic treatment is adequate. For instance, in one study addressing ventilator-associated pneumonia, the mean time for the complete resolution of fever was five days, for leukocytosis eight days, and for PaO^2^/FiO^2^ six days [81].

Monitoring clinical symptoms after antibiotic initiation is also relevant for evaluating adverse events. β-lactams might be associated with de novo gastrointestinal symptoms, changes in urine output, and neurological status, which are critical alarms for detecting undesirable effects [3].

### 3.2. Inflammatory Biomarkers

Inflammatory biomarkers have long been used to assess response to antibiotic treatment [82]. C-reactive protein (CRP) is produced in the liver in response to interleukin (IL)-6. It is measured daily in many intensive care units and frequently in hospitalized patients. It is often used in clinical practice to evaluate response to antibiotic treatment. A decrease above 50% after 48 h of antibiotic treatment in septic patients was associated with improved prognosis [83]. It has also been evaluated as a tool to help guide the duration of antibiotic therapy. In a meta-analysis including three randomized control trials, a CRP-based protocol was associated with a shorter duration of antibiotic treatment (minus 1.82 days), with no harm associated [84].

Procalcitonin has faster kinetics than CRP, and when negative, it may be safe to stop antibiotic treatment in patients with pneumonia [85]. Its kinetics are also helpful in complex inflammatory settings, such as in patients with pancreatitis [86].

Pancreatic stone protein (PSP) is another marker also shown to help monitor response to antibiotic treatment, although its kinetics in different infections are still less well-known [87].

**Figure 5:**
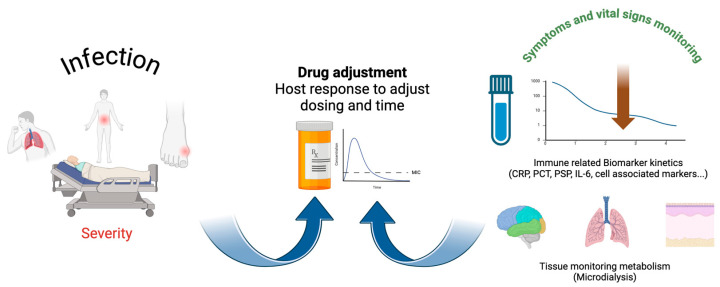
Dose adjustment should be a dynamic process, revised daily, including data from the host and biomarkers. Artificial intelligence and microdialysis may help to improve antibiotic prescription. CRP–C-reactive protein; PCT–Procalcitonin; IL 6-Interleukin 6. Created in BioRender.com with permission.

Overall, the levels of inflammatory biomarkers are expected to decrease in the days after antibiotic treatment. The complete integration and interpretation of data from several biomarkers simultaneously, including cell-associated biomarkers [88], in relation to antibiotic levels might soon become possible with AI methods (Figure 5). 

### 3.3. Microdialysis Is the Window into the Place Where the Infection Is

Microdialysis allows access to tissues. This technique uses microcapillaries and microfluidic techniques to measure different molecules in a specific tissue [89]. It has reached clinical use in multimodal neurological monitoring after traumatic brain injury, allowing metabolism to be monitored at a tissue level, in the context of secondary ischemia after major trauma. In addition to being relevant in direct tissue damage, it can also evaluate infection resolution at a tissue level. 

Microdialysis in adipose tissue has been used in septic patients as a surrogate of microcirculation. By measuring lactate, pyruvate, glucose, and glycerol levels, tissue metabolism and the response to infection were estimated. Upon improvement, the pyruvate–lactate ratio increased, and glycerol levels decreased.

Microdialysis was recently tested in a pilot study of secondary peritonitis, to help predict complications after surgery. The microcatheter was placed in the peritoneal space, measuring fluid glucose, lactate, pyruvate, and glycerol [90]. Interestingly, secondary peritonitis due to upper lesions was associated with higher glycerol levels, irrespective of further complications. The pattern of host response to infection is still unclear, in terms of either improvement or deterioration, but microdialysis might be fundamental to increasing biomarker specificity. 

In addition, this technique has been used to monitor antibiotic tissue concentrations. This might be relevant in difficult-to-treat infections such as diabetic foot [91] or to monitor tissue antibiotic concentration after non-parenteral administration [92]. Lung microdialysis might be helpful after antibiotic nebulization and can reveal local tissue inflammation during pneumonia, but this is still only being tested in animal models [93].

The use of artificial intelligence for simultaneous interpretation of antibiotic dosing and specific serum and tissue response patterns at 24–48 h will help judge a “good” response to antibiotics and match antibiotic dosing to an overall inflammatory response.

## 4. The Use of Machine Learning to Improve Antibiotic Dosing

The current limitations of traditional PK/PD models in fully capturing the intricacies and variations of patient responses highlight the need for more advanced and adaptable modeling approaches [94]. These models primarily focus on population-averaged parameters, which may not accurately represent individual patient dynamics. Critically ill patients, for example, often experience significant changes in drug metabolism and distribution due to varying physiological states, making it challenging to optimize dosage using conventional methods [95]. New models, such as digital twins, are emerging to address the problem of diseases with high morbidity and mortality rates [96]. These diseases have complex and multifactorial causes involving intricate molecular pathways and environmental factors that can affect the effectiveness of treatment. Digital twins create virtual models of individual patients to represent the unique and complex interactions between genetic, metabolic, and environmental factors. This approach could help select highly effective personalized therapies tailored to the specific molecular mechanisms of the disease within the patient’s unique metabolic, immune, and behavioral ecosystem. Each virtual twin could be treated with various drugs to predict specific patient responses based on numerous variables [97]. However, digital twins for PK/PD models in antibiotic treatment are still awaited.

### 4.1. Machine Learning

AI encompasses the theory and development of computer systems capable of performing tasks that typically require human intelligence, such as speech recognition, visual perception, decision-making, and language translation. Modern AI is underpinned by machine learning (ML) methods, which have been in existence since the 1940s [98]. Recent data digitalization, storage, and processing advancements have empowered these methods to propel contemporary AI initiatives. ML algorithms can be trained on datasets to make predictions through statistical modeling [99]. Furthermore, deep learning, a subset of ML, employs multi-layer computational models to discern intricate patterns in datasets by adjusting the machine’s internal parameters based on information processing in each layer [100].

**Figure 6:**
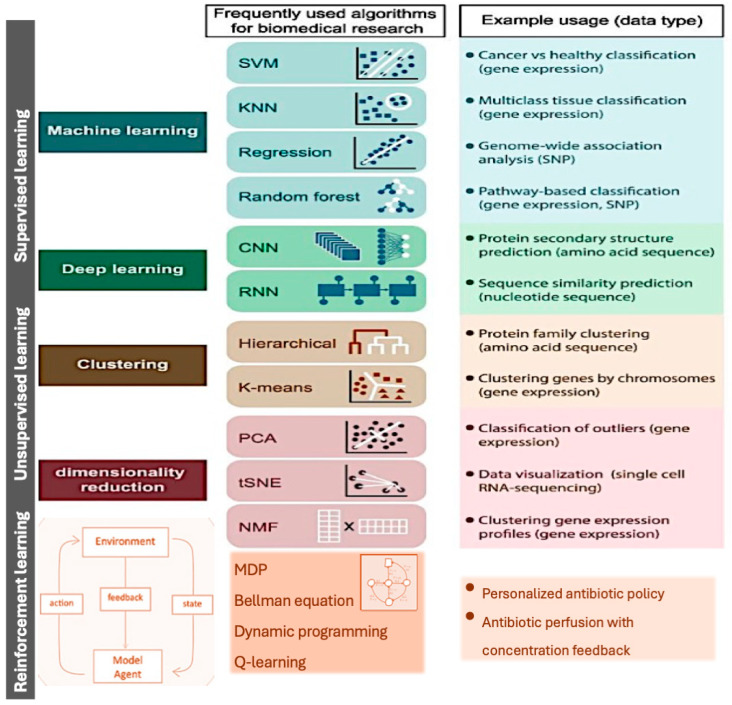
Different supervised, unsupervised, and reinforcement AI learning models/tools. Adapted from reference [104]. Abbreviations: SVM, sector vector machine; KNN, k-nearest neighbor; CNN, convolutional neural network; RNN: recurrent neural network; PCA, principal component analysis; tSNE, t-distributed stochastic neighbor embedding; NMF, non-negative matrix factorization; MDP, Markov decision process.

The field of ML encompasses a variety of algorithms, which can be broadly categorized into three main types: supervised learning (SL), unsupervised learning (UL), and reinforcement learning (RL) [101]. Figure 6 displays some examples of SL, UL, and RL.

SL involves creating predictive models based on training data with known input-output pairs. A human supervisor provides the algorithm with training datasets and an answer key, allowing the machine to validate its accuracy and learn. This type of learning is commonly employed for making predictions and includes algorithms such as random forest (RF), support vector machines (SVMs), and neural networks (NNs) [101]. RF is an ensemble learning method integrating multiple decision trees to enhance predictive accuracy and identify critical predictors of antibiotic efficacy [102]. RF effectively handles high-dimensional data and complex interactions. SVMs are effective for high-dimensional datasets and leverage the kernel trick to manage non-linear relationships, making them suitable for complex clinical data analysis [103]. NNs employ deep learning architectures and model intricate, non-linear interactions crucial for predicting clinical outcomes from PK/PD data [100]—Figure 6.

Unsupervised learning involves the use of a pattern-seeking algorithm that explores unlabeled data to discover associations that cannot be determined easily. The training data are unlabeled, so the machine must discern patterns without user input or an answer key [105]. Techniques like k-means and hierarchical clustering facilitate dimensionality reduction and the identification of patient subgroups with similar PK/PD profiles, aiding personalized treatment strategies. Methods such as principal component analysis and t-distributed stochastic neighbor embedding (t-SNE) reduce data complexity while preserving essential information, which is critical for interpreting high-dimensional clinical data, such as therapeutic response to therapy [106].

RL balances the methods of SL and UL to make predictions and discern patterns, where a virtual agent is trained to learn an optimal set of rules to maximize some reward. RL frameworks can adaptively refine dosing strategies using real-time patient data, allowing dynamic adjustments to treatment regimens as patient conditions evolve [107].

### 4.2. Selection of Data for Machine Learning Models

In the context of the uncomplicated ML process, four key elements collaboratively contribute to yielding outcomes: datasets (comprising raw data), preprocessing (an indispensable component of the process, ensuring successful outcomes), algorithms (interpreting the data), and selected features (the variables chosen from the dataset for analysis).

ML methods typically necessitate substantial volumes of data, routinely sourced from various outlets, encompassing the tracking of physiological data and frequent evaluation of vitals, laboratory values, and medications. A pivotal requirement for developing ML models is access to high-quality, dependable data alongside a clearly formulated and pertinent clinical or operational question that ML methods can address with the available data. The dissemination of patient health information is subject to stringent regulation owing to privacy concerns. Despite these challenges, the sharing of raw data has exhibited an increased prevalence in the biomedical literature, rising from less than 1% in the early 2000s to 20% at present [108]. Thought leaders have suggested expounding on significant data principles, such as privacy, transparency, and ethical standards, to create open, collaborative learning environments whereby de-identified data can be shared between researchers [109,110]. Data preprocessing encompasses procedures designed to transform raw data into actionable datasets. The primary objective of preprocessing is to evaluate and enhance the quality of the data to ensure their reliability for statistical analysis. A significant portion of publicly available research data is derived from electronic health records, which are predominantly compiled for clinical and billing purposes rather than specific research endeavors. This results in the availability of substantial volumes of data for analysis [111], as well as partially lost or unavailable (missing) data [112]. Other consequences may include incorrect value insertion and non-homogeneous records (different data types, scales, etc.) [111]. The process involves data cleaning to remove outliers, normalization to standardize variable ranges, and feature extraction to identify relevant variables influencing PK/PD outcomes [113,114,115]. As demonstrated by Yan et al. [94], integrating clinical data from diverse sources, such as electronic health records and laboratory results, enhanced the robustness of ML models. In a recent study, a hierarchical approach was employed to extract variables from electronic health records to detect antibiotic exposure retrospectively [116]. Using an RF model, multiple ML models were developed and trained to evaluate the incremental values of numerous variables, including demographics, comorbidities, medications, laboratory data, and vital signs. This model, predicated on decision trees, was able to capture nonlinear interactions without the need for prior specification. The ultimate model accurately quantified instances of exposure and duration of therapy, demonstrating a mean area under the curve of 0.85 and a mean error of 1.0 days of therapy. These pre-processing procedures and outlier removal are estimated to represent 60% of resource effort in predictive risk modeling [117].

When determining the suitable ML modality, it is essential to be guided by a specific inquiry, considering the adequacy and accessibility of data and the availability of a robust computational infrastructure. For instance, in the context of identifying optimal dosing protocols for medications with frequently fluctuating dosing parameters, such as β-lactams, RL methodologies may prove most effective [118]. Other examples using deep learning-based methods suitable for dealing with time series include recurrent neural networks for diagnostic prediction [117,119] and more recently, gated recurrent units for early prediction of septic shock [120]. Implementing certain ML approaches in healthcare settings is constrained by the substantial computational demands and the requisite processing power, which are frequently absent in many hospital facilities. It is constructive to note, however, that these challenges primarily pertain to the initial model training phase, as opposed to the subsequent utilization of the developed models.

Comprehending the efficacy of ML models in ascertaining their clinical applicability presents challenges. The incorporation of human factor principles, which positively influence human interaction with ML tools, alongside considerations regarding design, workflow, and changes in management, constitute vital components of implementation science driving the adoption of ML solutions [109]. Collaboration between physicians and data scientists is paramount to achieve successful and helpful ML solutions [121].

The reliability, reproducibility, and transparency of studies utilizing ML methods pose concerns for experts and physicians. A pivotal issue revolves around the potential amplification of low-level signals lacking clinical relevance in these studies, as well as their generalizability beyond the source database [122]. Some advances have been made to achieve higher reproducibility and transparency in this research [123,124]. Numerous frameworks and platforms exist to make AI research more transparent and reproducible, such as for sharing code (Bitbucket, GitHub, and GitLab, among others) [125].

### 4.3. Application of Machine Learning Models to Antibiotic Pharmacokinetics and Pharmacodynamics

The application of ML models has demonstrated significant and reassuring improvements over traditional PK/PD models in predicting clinical outcomes. Table 1 describes some of the studies on PK/PD ML algorithms. RF models have shown significant capabilities in identifying critical predictors of antibiotic efficacy and optimizing dosing regimens. An example of this application was reviewed by Peiffer-Smadja et al. [126]. An RF model was employed to identify key predictors influencing the effectiveness of antibiotic therapy and to suggest optimal dosing, achieving significantly higher predictive accuracy than traditional models. Critical factors such as specific biomarkers and microbial resistance profiles were identified as key predictors. The model provided individualized dosing recommendations that improved therapeutic outcomes (Table 1).

SVMs have also proven effective in identifying optimal antibiotic dosing regimens that optimize patient outcomes. Kim et al. describe the application of SVMs in predicting patient responses to antibiotic dosing in cases of multidrug-resistant bacterial infections [139]. Also, SVM accurately distinguished patients who achieved favorable outcomes and those who did not, independently of the prespecified PK/PD targets [140]. Neural networks showcased superior performance in modeling the dynamic interactions affecting therapeutic success, particularly in scenarios with multifactorial influences such as comorbidities [95]. Komorowski et al. highlighted the use of deep learning models to optimize antibiotic dosing in critically ill patients [118]. The neural network models achieved higher predictive accuracy than traditional PK/PD models. The deep learning approach effectively captured complex interactions within the data, indicative of optimal dosing strategies. Explainable artificial intelligence techniques, such as Shapley Additive Explanations (SHAP), have advanced the interpretability of these models. SHAP values provide insights into the most influential factors driving clinical outcomes, fostering clinician trust and paving the way for practical implementation [141,142].

Adherence to regulatory and ethical standards is crucial for the safe and effective implementation of advanced ML models in clinical practice. Topol highlighted the convergence of human and artificial intelligence, emphasizing the need for stringent oversight to ensure patient safety [143]. Integrating real-time data from wearable devices and electronic health records offers opportunities for continuous, personalized treatment adjustments [144].

Online-accessible prediction models are already available for assessing the likelihood of beta-lactam antibiotics being ineffective in ICU patients within 12–36 h of starting the treatment [145]. These models use commonly available patient information and help to optimize the use of antibiotics. The models for predicting beta-lactam antibiotic ineffectiveness were created and verified using RF, logistic regression (LR), and naïve Bayes (NB) techniques with data from 376 patients. An additional assessment with 150 ICU patients was conducted for external validation. The performance was evaluated by examining discrimination, calibration, and net benefit at the standard probability threshold of 0.20. Age, gender, serum creatinine levels, and the type of beta-lactam antibiotic were identified as predictors of beta-lactam antibiotic ineffectiveness. In the external validation, the RF, LR, and NB models demonstrated strong discrimination, with an area under the curve of 0.79 [95% CI 0.72–0.86], 0.80 [95% CI 0.73–0.87], and 0.75 [95% CI 0.67–0.82], respectively, and showed net benefits with the RF and LR models.

In addition to this, there are already some promising ML models available to optimize drug dosing. OptiDose [146] calculates the best individualized dosing plan for pharmacokinetic–pharmacodynamic models in different scenarios with various methods of administration, by solving an optimal control problem. The goal is to calculate a control that brings the system as close as possible to a desired reference function by minimizing the cost. In pharmacokinetic–pharmacodynamic modeling, the controls are the administered doses, and the reference function can be the disease progression. Drug administration at specific time points provides a finite number of discrete controls, the drug doses, determining the drug concentration and its effect on disease progression. Consequently, rewriting the cost gives a finite-dimensional optimal control problem that depends only on the doses. Adjoining techniques allow the efficient computation of the gradient of the cost. This allows the solving of the optimal control problem with robust algorithms, such as quasi-Newton methods from finite-dimensional optimization. However, this approach still requires validation on antibiotic regimens.

Another model is the whole-body physiologically based pharmacokinetic model, which compares the killing of bacteria with different antibiotic susceptibility [147]. This is valuable for drug development and the optimal use of antibiotics. In this context, it was applied to ciprofloxacin for ICU patients and, based only on plasma concentration data, tissue and organ concentration time profiles in patients were predicted using the developed model. The bacterial killing was predicted to be most efficient in the lungs and kidneys, which aligned well with ciprofloxacin’s indications for pneumonia and urinary tract infections. Furthermore, a function based on available information on bacterial killing by the immune system in vivo was incorporated.

Adherence to regulatory and ethical standards is crucial for the safe and effective implementation of advanced ML models in clinical practice.

In conclusion, ML has the potential to bridge the gap between achieving PK/PD targets and realizing clinical outcomes. ML can significantly enhance therapeutic efficacy and patient outcomes by improving predictive power and tailoring antibiotic dosing more precisely. Continued research, improvements in data quality, and interdisciplinary collaboration will be vital for making the most of these innovations, ultimately improving patient care and combating antibiotic resistance.

## 5. Conclusions

In this review, we have discussed the challenges of β-lactam dosing. Although the use of TDM may be tempting, limitations arise due to the poor understanding of intra and inter-individual variability. Furthermore, differences in PD may arise from bacteria and host individual characteristics. Embracing complexity with AI may facilitate the understanding of individual clinical pathways to foster better antibiotic selection and dosing.

## Figures and Tables

**Table 1:** Algorithms used for the development of AI models for various PK/PD studies, along with their advantages and limitations.

Algorithm	Aim/Target/Study	Advantages	Limitations
Bayesian/WinBUGS version 1.4	To handle data below the limit of quantification [127]	Prior information from the literature can be used directly for model-fitting; easy implementation.	Long computational time. Negative data in certain PK/PD models, which are not possible.
Bayesian/PKBUGS (version 1.1)/WinBUGS (version 1.3)	Pharmacokinetic analysis of sirolimus concentration data for therapeutic drug monitoring [128]	Easy incorporation of prior information with current data; identification of possible covariate relationship.	A limited number of datasets and poorly informative data.
Support Vector Machine/Least-Square SVM	Drug concentration analysis of sample drug based on individual patient profile [129]	Personalized model for every new patient. SVM-based approaches are more accurate than the PK modeling method for predicting drug concentration.	Outliers in samples greatly affect the model, limiting its accuracy.
Support Vector Machine/Drug Administration Decision Support System (DADSS) and Random Sample Consensus (RANSAC)	Prediction of drug concentration, ideal dose, and dose intervals for a new patient [130]	More flexible and structurally adjustable.	The noise of the dataset impacts the overall predictivity of the algorithm.
Support Vector Machine/Random Forest Model/K means	A predictive model was developed and validated to distinguish Enterococcus faecium vancomycin-resistant strains using SVM, K means, and random forest (RF) [131]	Overall good classification performances for the isolates from the specimens, with mean accuracy, sensitivity, and specificity of 0.78, 0.79, and 0.77.	Susceptibility results must be confirmed by routine methods.
Support Vector System + Random Forrest Model	Pharmacodynamic drug interaction (PDI) based on side-effect similarity (SES), Chemical Similarity (CS), and target protein connectedness (TPC) [132]	PDI was predicted with an accuracy of 89.93% and an AUC value of 79.96%.	Requires more data processing and filtration.
Linear Regression (LASSO)/Gradient Boosting Machines/ XGBoost/Random Forest	Prediction of the plasma concentration-time series and area under the concentration vs. time curve from 0 to 24 h after repeated dosing of rifampicin [133]	Time-efficient analysis; improved method for covariate selection.	Risk of results not being clinically relevant.
XGBoost	Joint multilayer perceptron (JointMLP), a new deep-learning model for predicting vancomycin therapeutic drug monitoring (TDM) levels, comparing its performance with population pharmacokinetic models, extreme gradient boosting (XGBoost), and TabNet [134]	JointMLP model outperformed other models in predicting vancomycin TDM levels in internal and external datasets.	Further research is needed to compare the AUC/MIC range with this approach.
Simulated Annealing k-Nearest-Neighbor (SA-kNN)/Partial Least-Square (PLS)/Multiple Linear Regression (MLR)/Sybyl version 6.7	Prediction of pharmacokinetic parameters of antimicrobial agents in humans, based on their molecular structure [135]	Cost-effective; requires smaller sample size.	Requires multiple model-generation methods. Interpretation of individual descriptors is almost impossible.
Drug Target Interaction Convolutional Neural Network (DTICNN)	Identification of the drug–target interactions and predict potential drug molecules [136]	Cost-effective; time-saving	Large datasets are required.
Deep Long Short-Term Memory (DeepLSTM)	Computational methods to validate the interaction between drugs and target [137]	Based on position-specific scoring matrix (PSSM) and Legendre moment (LM) (drug molecular substructure fingerprints).	Large datasets are required.

Adapted from reference [138].

## Data Availability

No new data were created or analyzed in this study.
